# Urine Neutrophil Gelatinase-associated Lipocalin (NGAL) for Prediction of Persistent AKI and Major Adverse Kidney Events

**DOI:** 10.1038/s41598-020-65764-w

**Published:** 2020-05-26

**Authors:** Nuttha Lumlertgul, Monpraween Amprai, Sasipha Tachaboon, Janejira Dinhuzen, Sadudee Peerapornratana, Stephen J Kerr, Nattachai Srisawat

**Affiliations:** 1 Division of Nephrology, Faculty of Medicine, Chulalongkorn University, Bangkok, Thailand; 2 Excellence Center for Critical Care Nephrology, King Chulalongkorn Memorial Hospital, Bangkok, Thailand; 3 Critical Care Nephrology Research Unit, Faculty of Medicine, Chulalongkorn University, Bangkok, Thailand; 4 Department of Laboratory Medicine, Faculty of Medicine, Chulalongkorn University, Bangkok, Thailand; 5 The Center for Critical Care Nephrology, CRISMA, Department of Critical Care Medicine, University of Pittsburgh School of Medicine, Pittsburgh, Pennsylvania USA; 6 Research Affairs, Faculty of Medicine, Chulalongkorn University, Bangkok, Thailand; 7 The HIV Netherlands Australia Thailand Research Collaboration (HIV-NAT), The Thai Red Cross AIDS Research Centre, Bangkok, Thailand; 8 The Kirby Institute, The University of New South Wales, Sydney, Australia; 9 Academic of Science, Royal Society of Thailand, Bangkok, Thailand; 10 Tropical Medicine Cluster, Chulalongkorn University, Bangkok, Thailand; 11 Excellence Center for Critical Care Medicine, King Chulalongkorn Memorial Hospital, Bangkok, Thailand

**Keywords:** Biomarkers, Diseases, Medical research, Nephrology

## Abstract

We aimed to determine whether urinary neutrophil gelatinase-associated lipocalin (uNGAL) can accurately predict persistent AKI, major adverse kidney events at 30 days (MAKE30) and 365 days (MAKE365) in hospitalized AKI patients. This is a retrospective study of adult patients who were admitted at King Chulalongkorn Memorial Hospital. We performed multivariable logistic regression for persistent AKI, MAKE30, and MAKE365. We developed equations for predicting MAKE30 and MAKE365 and divided the dataset into derivation and validation cohorts. uNGAL performance and predictive models were assessed using the area under the receiver operating characteristic curve (AROC). Among 1,322 patients with AKI, 76.9%, 45.1%, and 61.7% had persistent AKI, MAKE30, and MAKE365. The AROC were 0.75 (95% confidence interval[CI] 0.70–0.80), 0.66 (95%CI 0.61–0.71), and 0.64 (95%CI 0.59–0.70) for prediction of persistent AKI, MAKE30, and MAKE365 by uNGAL. The AROC in the validation dataset combining uNGAL with clinical covariates were 0.74 (95%CI 0.69–0.79) and 0.72 (95%CI 0.67–0.77) for MAKE30 and MAKE365. We demonstrated an association between uNGAL and persistent AKI, MAKE30, and MAKE365. Prediction models combining uNGAL can modestly predict MAKE30 and MAKE365. Therefore, uNGAL is a useful tool for improving AKI risk stratification.

## Introduction

Acute kidney injury (AKI) is reported in 52.9–57.3% of critically ill patients and strongly associated with increased morbidity and mortality^1–3^. In addition, AKI survivors have increased risk of developing chronic kidney disease (CKD), CKD progression, and end-stage kidney disease (ESRD). Factors associated with an increased risk of long-term complications include diabetes, older age, number of AKI episodes, AKI severity and serum albumin^4–6^. Recently, AKI duration was ascertained as another prognostic factor for short-term and long-term mortality^7–10^.

Novel biomarkers measured at the time of intensive care unit (ICU) admission have been reported to predict short-term and long-term outcomes. Examples include urinary neutrophil gelatinase-associated lipocalin (uNGAL), urinary interleukin-18 (IL-18), urinary kidney injury molecule-1 (KIM-1), urinary insulin-like growth factor-binding protein-7 and tissue inhibitor of metalloproteinases-2 (TIMP-2xIGFBP-7)^11–13^. uNGAL is a 25-kDa protein belonging to the lipocalin family. uNGAL is produced in renal epithelia and leukocytes in response to tubular injury and systemic inflammation. High uNGAL can be used to predict AKI^14–17^, discriminate intrinsic AKI from pre-renal AKI^18,19^, predict renal non-recovery, in-hospital mortality^20–22^, long-term CKD progression, ESRD, and mortality^11,12,23^. Previous predictive models have utilized uNGAL to predict AKI, in-hospital renal replacement therapy (RRT), or death^21,24,25^. However, no studies have assessed the clinical utility of uNGAL for assessment of overall spectrum of outcomes ranging from in-hospital mortality, renal replacement therapy (RRT), and renal non-recovery, to survival and renal function after hospital discharge.

King Chulalongkorn Memorial Hospital has employed uNGAL test as a routine measurement in AKI patients since 2015. This study aimed to first evaluate the role of uNGAL in an adult population for predicting persistent AKI. Secondly, we aimed to assess and evaluate, using multivariable logistic regression, the usefulness of uNGAL measurement in combination with standard clinical covariates for prediction of 30-day and 365-day major adverse kidney events in a heterogeneous adult population.

## Results

### Population

**Figure 1:**
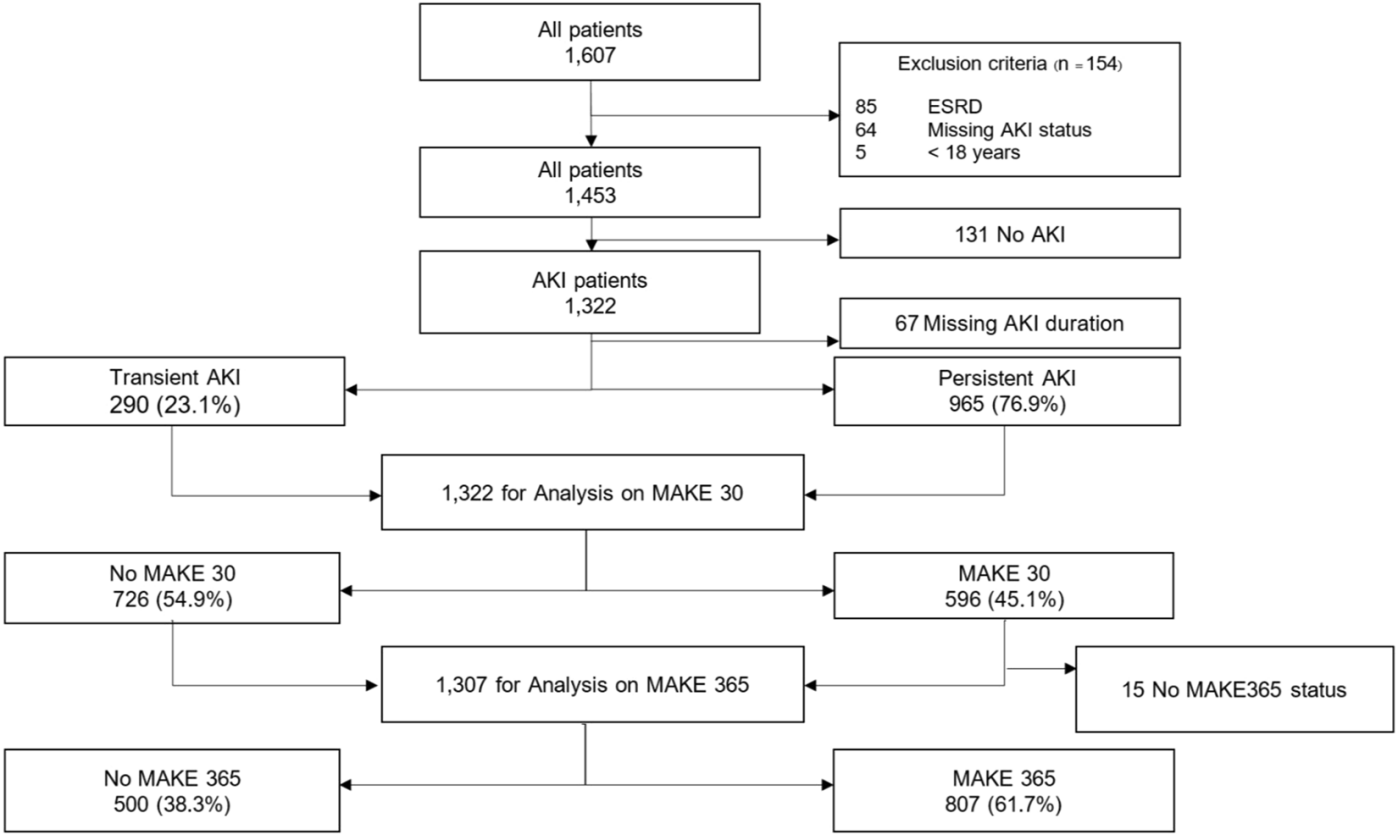
Study flow chart.

During the study period, there were 1,607 patients with at least one uNGAL measurement; after excluding duplicates, 1,322 AKI patients were included in the analysis. In our study, baseline SCr was obtained from pre-admission values in 947 (71.6%) patients, the majority of patients in our study. We used MDRD equation back-estimation in 5.6% and the first SCr on admission in 22.8%. Among AKI patients, 23.1% were detected with transient AKI and 76.9% with persistent AKI. At 30 days, MAKE30 occurred in 45.1%. At 365 days, 1,307 patients were available for MAKE365 analysis; 61.7% had MAKE365 (Fig. 1).

Most patients were male (55%), and had a median age of 66 (IQR, 55–80) years. Baseline SCr was obtained from pre-admission values in 947 (71.6%) patients. We used MDRD equation back-estimation in 5.6% and the first SCr on admission in 22.8%. uNGAL was requested in the ward, ICU, and emergency department in 70.7%, 24.4%, and 4.9% of all patients, respectively. Indications for uNGAL request were presented in Supplementary Table 1.

### Urine chemistry and uNGAL for prediction of persistent versus transient AKI

Most of the AKI population (76.9%) had persistent AKI. These patients presented more frequently with AKI stage 3, sepsis, CKD, had a higher median SCr at diagnosis, and a higher peak SCr. They were less likely to have AKI from ischemic cause compared with transient AKI patients (p < 0.001). Those with persistent AKI had a higher proportion of MAKE30 and MAKE365, including their individual components. At 30 days, renal recovery occurred in 55.3% of those with persistent AKI versus 77.6% of those with transient AKI (p < 0.001) (Table 1).

**Table 1:** Comparison between urine NGAL, FENa, FEuric, and FEurea in patients with transient AKI versus persistent AKI.

Variables	Total (N = 1255)	Transient AKI (N=290)	Persistent AKI (N=965)	p-value
Age, years (mean ± SD)	66.11 ± 17.27	65.13 ± 17.93	66.40 ± 17.06	0.27
Male (%)	691 (55.1)	163 (56.2)	528 (54.7)	0.65
Place of NGAL sent (%)				0.14
Ward	878 (70.2)	217 (74.8)	665 (68.9)	
ED	60 (4.8)	13 (4.5)	47 (4.9)	
ICU	313 (25.0)	60 (20.7)	253 (26.2)	
Admission diagnosis (%)				0.16
Cardiology	256 (20.5)	67 (23.2)	189 (19.7)	
Infection	296 (23.7)	64 (22.2)	232 (24.2)	
Malignancy	162 (13.0)	37 (12.8)	125 (13.0)	
Trauma/Surgery	60 (4.8)	12 (4.2)	48 (5.0)	
Rheumatology	27 (2.2)	5 (1.7)	22 (2.3)	
Hematology	51 (4.1)	9 (3.1)	42 (4.4)	
Endocrinology	24 (1.9)	7 (2.4)	17 (1.8)	
Ob-Gyn	4 (0.3)	2 (0.7)	2 (0.2)	
Gastroenterology	104 (8.3)	25 (8.7)	79 (8.2)	
Genitourinary system	72 (5.8)	26 (9)	46 (4.8)	
Respiratory system	163 (13.1)	28 (9.7)	135 (14.1)	
Others	29 (2.3)	7 (2.4)	22 (2.3)	
Admission type (%)				0.19
Medical	1,019 (81.7)	237 (82.0)	782 (81.5)	
Elective surgery	129 (10.3)	35 (29.9)	94 (9.8)	
Emergency surgery	100 (8.0)	17 (5.9)	83 (8.7)	
AKI staging (%)				<0.001
1	443 (35.3)	172 (59.3)	271 (28.1)	
2	279 (22.2)	75 (25.9)	204 (21.1)	
3	533 (42.5)	43 (14.8)	490 (50.8)	
Sepsis (%)	546 (43.5)	100 (34.5)	446 (46.2)	<0.001
Ischemic cause (%)	672 (53.6)	187 (64.5)	485 (50.4)	<0.001
Nephrotoxic AKI (%)	113 (9.0)	26 (9.0)	87 (9.0)	1.00
CKD (%)	533 (42.5)	90 (31.0)	443 (45.9)	<0.001
Comorbidities (%)				
Diabetes	463 (36.9)	107 (36.9)	356 (36.9)	1.00
Hypertension	660 (52.6)	156 (53.8)	504 (52.2)	0.69
Congestive heart failure	108 (8.6)	30 (10.3)	78 (8.1)	0.23
Ischemic heart disease	55 (4.4)	9 (3.1)	46 (4.8)	0.23
Chronic liver disease	196 (15.6)	44 (14.9)	152 (15.8)	0.81
Baseline Cr, mg/dL	1.5 (1.01, 2.5)	1.2 (0.9,1.7)	1.6 (1.1, 2.7)	<0.001
Baseline GFR, mL/min/1.73 m^2^	45.5 (27.8, 70.3)	55.8 (39.5,73.8)	42.9 (25.6, 68.9)	<0.001
Cr at AKI diagnosis, mg/dL	2.3 (1.6, 3.4)	1.9 (1.5,2.6)	2.6 (1.9, 3.8)	<0.001
Peak creatinine, mg/dL	2.9 (1.9, 4.3)	1.8 (1.4, 2.5)	3.3 (2.5, 5)	<0.001
Last GFR at day 30, mL/min/1.73 m^2^	43.2 (27.7, 65.5)	58.7 (41.1,79.6)	38.7 (23.7,57.4)	<0.001
Urine NGAL, ng/mL	376 (102, 1500)	103 (72, 273)	603 (124, 2004)	<0.001
Urine NGAL-Cr ratio, ng/mg	5.5 (1.8, 32.9)	1.7 (0.8,4.9)	8.9 (2.5,42.3)	<0.001
FENa, %	1.7 (0.6, 3.9)	1.1 (0.5, 2.1)	2.1 (0.7, 5.1)	<0.001
FEUric, %	12.4 (6.3, 22.1)	10.6 (5.7, 14.7)	13.5 (6.5, 24.7)	0.001
FEUrea, %	38.4 (24.0, 50.8)	40.3 (26.9, 49.4)	36.9 (23.3, 51.6)	0.67
MAKE30 (%)	573 (45.7)	70 (24.1)	503 (52.1)	<0.001
MAKE365 (%)	774 (62.4)	113 (39.4)	661 (69.3)	<0.001
30-day mortality (%)	358 (28.5)	43 (14.8)	315 (32.6)	<0.001
365-day mortality (%)	634 (50.5)	99 (34.4)	535 (56.1)	<0.001
30-day RRT (%)	269 (21.4)	23 (7.9)	246 (25.5)	<0.001
365-day RRT (%)	308 (24.5)	29 (10)	279 (28.9)	<0.001
Doubling of Cr at day 30 (%)	218 (17.4)	25 (8.6)	193 (20)	<0.001
Doubling of Cr at day 365 (%)	911 (72.6)	161 (55.5)	750 (77.7)	<0.001
Recovery at day 30 (%)	759 (60.5)	225 (77.6)	534 (55.3)	<0.001
Recovery at day 365 (%)	398 (31.7)	124 (42.7)	274 (28.4)	<0.001
Length of hospital stay, days	17 (9, 32)	13 (6,25)	19 (10,36)	<0.001

Values in cell is median (IQR).

Abbreviations NGAL, neutrophil gelatinase-associated lipocalin; FENa, fractional excretion of sodium; FEuric, fractional excretion of uric; FEurea, fractional excretion of urea; Cr, creatinine; AKI, acute kidney injury; SD, standard deviation; ED, emergency department; ICU, intensive care unit; CKD, chronic kidney disease; GFR, glomerular filtration rate; MAKE, major adverse kidney events; RRT, renal replacement therapy.

**Figure 2:**
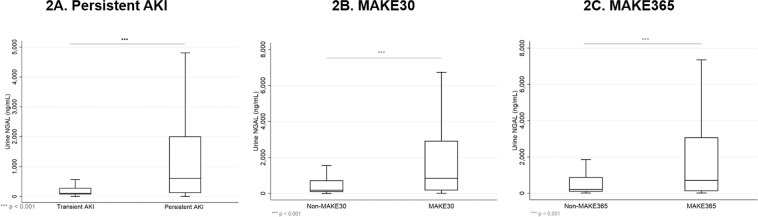
Box plot showing uNGAL concentration distribution between persistent versus transient AKI (**A**), MAKE30 versus non MAKE30 (**B**), and MAKE365 versus non-MAKE365 (**C**). The box represents the 25^th^ and 75^th^ percentiles and line within the box represents the median. Pairwise comparison was calculated by Mann-Whitney U test.

Median uNGAL concentrations in persistent AKI were 603 (IQR 124, 2004) ng/mL versus 103 (IQR 72,273) ng/mL in transient AKI (p < 0.001, Fig. 2A). Univariate logistic regression analysis revealed AKI staging, sepsis, chronic kidney disease, FENa, FEuric, and log_e_ uNGAL to be associated with persistent AKI (p < 0.001), while ischemic cause was a protective factor (p < 0.001). After adjusting for these covariates in a multivariate logistic regression analysis, log_e_ uNGAL remained significant (Table 2).

**Table 2:** Univariate and multivariate logistic regression analysis for persistent AKI.

	Univariate analysis		Multivariate analysis	
	OR (95% CI)	P value	OR (95% CI)	P value
Log uNGAL	1.83 (1.67,2.06)	<0.001	1.72 (1.42,2.08)	<0.001
Log uNGAL-Cr	1.71 (1.54,1.91)	<0.001		
ICU vs other wards	1.36 (0.99,1.87)	0.06	2.09 (1.08,4.04)	0.03
**AKI stage**				
1	Reference	0.001	Reference	0.10
2	1.73 (1.25,2.39)	<0.001	1.69 (0.90,3.20)	0.003
3	7.23 (5.02,10.43)		3.21 (1.50,6.85)	
Sepsis	1.63 (1.24,2.15)	<0.001	0.96 (0.50,1.82)	0.90
Ischemic cause	0.56 (0.43,0.73)	<0.001	0.68 (0.36,1.28)	0.23
CKD	1.89 (1.43,2.49)	<0.001	1.38 (0.73,2.62)	0.33
Cr at AKI diagnosis	1.47 (1.31,1.66)	<0.001	2.23 (1.50,3.32)	<0.001
FENa	1.17 (1.08,1.26)	<0.001	1.17 (1.03,1.32)	0.01
FEuric	1.04 (1.02,1.06)	<0.001	1.01 (0.98,1.03)	0.69

Abbreviations OR, odds ratio; CI, confidence interval; uNGAL, urine neutrophil gelatinase-associated lipocalin; FENa, fractional excretion of sodium; FEuric, fractional excretion of uric; Cr, creatinine; CKD, chronic kidney disease.

**Figure 3:**
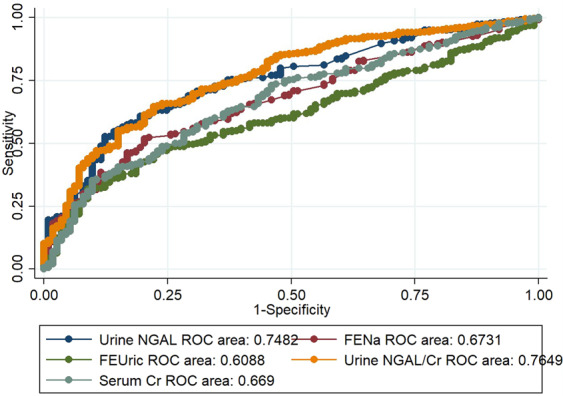
Area under the curve receiver operating characteristic curve for Urine NGAL, Urine NGAL to Cr ratio, FENa, FEuric, and Cr at AKI diagnosis for persistent AKI; NGAL, neutrophil gelatinase-associated lipocalin; FENa, fractional excretion of sodium; FEuric, fractional excretion of uric; Cr, creatinine; AKI, acute kidney injury.

To compare the performance of each individual biomarker in the prediction of persistent AKI, subjects who had missing values in one or more biomarkers were excluded for ROC curve analysis. Overall, there were 446 patients with available uNGAL, uNGAL/Cr, FENa, FEuric, and SCr collected on the same day. All studied biomarkers were significant predictors of persistent AKI. uNGAL/Cr ratio showed the best discrimination of persistent AKI versus transient AKI with an AROC of 0.76 (95% CI = 0.72–0.81), comparably to uNGAL (AROC 0.75, 95% CI = 0.70–0.80) but superior to other biomarkers (Fig. 3).

### MAKE outcomes at 30 days

The overall MAKE30 incidence was 45.1%. The incidence of each endpoint contributing to MAKE30 was 28.3% death, 12.0% RRT, and 4.8% persistent renal dysfunction. Median uNGAL concentration between patients with MAKE30 and without MAKE30 were 846 (IQR 176, 2907) versus 179 (IQR 96, 704) ng/mL, p < 0.001 (Fig. 2B). When comparing the performance of our multivariate logistic models for predicting MAKE30 and MAKE365, we randomized the entire dataset into training and validation datasets, representing 70% and 30% of the observations, respectively; patient characteristics between these groups comparable (Supplementary Table 2). In the training dataset, 417/925 patients developed MAKE30. Table 3 shows the results from the multivariate model for MAKE at 30 days. An increased risk of MAKE30 was associated with uNGAL concentration, male sex, patients in the ICU versus other wards, those with AKI stage 3 versus 2 and 1, with sepsis, ischemic cause of AKI, malignancy and chronic liver disease.

**Table 3:** Results from multivariable logistic regression model of factors associated with major adverse kidney events at 1 month (MAKE30) and 1 year (MAKE365).

	Multivariate model for MAKE30			Multivariate model for MAKE365		
	Coef.	aOR (95%CI)	P	Coef.	aOR (95%CI)	P
Intercept	−4.04			−3.77		
Log_e_ uNGAL concentration	0.38	1.47 (1.32–1.63)	<0.001	0.34	1.41 (1.26–1.57)	<0.001
Age, per 10 year increase	—	—	—	0.14	1.15 (1.06–1.26)	0.001
Male versus female	0.27	1.31 (0.97–1.77)	0.08	—	—	—
ICU vs other wards	0.63	1.88 (1.33–2.67)	<0.001	0.30	1.35 (0.93–1.97)	0.113
AKI stage 3 vs 2/1	0.86	2.35 (1.73–3.21)	<0.001	0.52	1.69 (1.21–2.34)	0.002
Sepsis	0.39	1.47 (1.04–2.08)	0.03	0.36	1.43 (1.03–1.98)	0.03
Ischaemic cause	0.32	1.37 (0.98–1.92)	0.06	—	—	—
Malignancy	0.61	1.85 (1.18–2.9)	0.007	0.75	2.13 (1.3–3.48)	0.003
Ischemic heart disease	—	—	—	1.90	6.69 (2.41–8.56)	<0.001
Persistent AKI	0.48	1.61 (1.06–2.43)	0.02	0.82	2.28 (1.55–3.35)	<0.001
Chronic liver disease	0.39	1.48 (0.98–2.23)	0.06	0.76	2.14 (1.38–3.34)	0.001

How to calculate the probability of the patient experiencing a major adverse kidney event within 1 month (MAKE30).

For example, a female with uNGAL concentration of 2000, from ICU, with stage 2 AKI, septic, non-ischaemic cause, a malignancy, chronic liver disease and persistent AKI: MAKE30 Score = −4.04 + (loge2000*0.38) + 0.63 + 0.39 + 0.61 + 0.48 + 0.39) = 1.348.

The exponential of 1.348 is 3.849, so the probability this person will develop MAKE30 is 3.849/(1+3.849) = 0.79 (79%).

**Figure 4:**
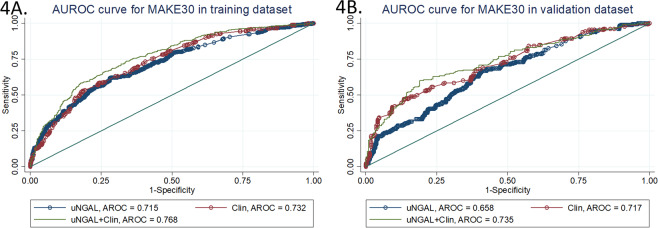
ROC curves from a multivariate clinical model, uNGAL, and the model with uNGAL for prediction of MAKE30 in the training dataset (**A**) and validation dataset (**B**).

We used the estimates from the multivariate logistic regression model to generate a MAKE30 predictive score for every individual in the training dataset. The overall mean score was 0.24 ± 1.11. For those who developed MAKE30 the mean was 0.33 ± 1.0 compared with −0.71 ± 0.98 in those who did not (mean difference 1.04 (95%CI 0.91–1.17); P < 0.001). The AROC from this model was 0.77 (95%CI 0.73–0.80), indicating a reasonable ability of the score to discriminate between those who developed MAKE30 and those who did not. Furthermore, this AROC was significantly better than a model which only included uNGAL concentration (AROC 0.72 (95%CI 0.68–0.75); P for difference in AROC < 0.001) (Fig. 4A, Table 4). We stratified patients according to deciles of scores; Table 5 shows the observed versus expected number of patients who developed MAKE30 in each stratum. The Hosmer-Lemeshow test statistic was 5.04 (P = 0.75), indicating no evidence for lack of fit (Table 5).

**Table 4:** AUC of clinical model with uNGAL for prediction of MAKE30 and MAKE365.

	AUC (95% CI)				
	uNGAL	Clinical model^a,b^	uNGAL + clinical model^a,b^	P value^c^	P value^d^
**MAKE30**					
Training cohort	0.72 (0.68–0.75)	0.73 (0.69–0.76)	0.77 (0.73–0.80)	<0.001	<0.001
Validation cohort	0.66 (0.61–0.71)	0.71 (0.66–0.76)	0.74 (0.69–0.79)	<0.001	<0.001
**MAKE365**					
Training cohort	0.70 (0.67–0.74	0.74 (0.71–0.78)	0.77 (0.74–0.80)	<0.001	<0.001
Validation cohort	0.64 (0.59–0.70	0.70 (0.65–0.75)	0.72 (0.66–0.77	<0.001	<0.001

^a^Clinical model for MAKE30 is comprised of participant sex, ICU setting, AKI staging, sepsis, ischemic AKI, malignancy, persistent AKI, and chronic liver disease.

^b^Clinical model for MAKE365 is comprised of age, ICU setting, AKI staging, sepsis, malignancy, persistent AKI, and chronic liver disease.

^c^P value AUC of biomarker + clinical model compared with uNGAL.

^d^P value AUC of biomarker + clinical model compared with clinical model.

**Table 5:** Expected and observed numbers of major adverse kidney events at 1 month (MAKE30) and 1 year (MAKE365), stratified by deciles of logistic regression score (1 = lowest, 10 = highest), in the training and validation datasets.

MAKE30	Training sample			Validation sample		
	Number of patients	Number of patients with MAKE30 expected	Number of patients with MAKE30 observed	Number of patients	Number of patients with MAKE30 expected	Number of patients with MAKE30 observed
1	94	10.9	11	40	5.3	8
2	90	16.9	13	39	8.7	11
3	91	22.8	25	40	11.4	11
4	91	29.4	34	39	13.6	13
5	92	36.2	34	39	16.2	15
6	91	42.6	41	40	18.7	12
7	92	50.9	52	39	20.5	21
8	91	58.3	61	40	24.5	26
9	92	67.2	62	39	27.3	26
10	91	76.8	79	39	31.9	35
Total	915	412	412	394	178.1	178
Hosmer-Lemeshow test statistic*			5.04, P=0.75			9.16, P = 0.52
AROC			0.77			0.74
**MAKE365**	**Number of patients**	**Number of patients with MAKE365 expected**	**Number of patients with MAKE365 observed**	**Number of patients**	**Number of patients with MAKE365 expected**	**Number of patients with MAKE365 observed**
1	91	18.2	18	39	10.9	13
2	91	31.9	34	39	16.1	13
3	91	41.3	42	39	19.5	18
4	91	49.4	48	39	22.0	21
5	90	55.5	56	39	24.0	27
6	91	62.0	59	39	25.8	26
7	91	67.6	65	39	27.5	27
8	91	73.1	76	39	29.3	32
9	91	77.9	82	39	31.5	30
10	90	83.1	80	38	33.5	33
Hosmer-Lemeshow test statistic*			4.73, P = 0.79			4.30, P= 0.93
C statistic			0.768 (95%CI 0.74–0.80)			0.717 (95%CI 0.67–0.77

^*^Follows a X^2^ distribution with 8 degrees of freedom for the training sample and 10 degrees of freedom for the validation sample.

When the same predictive score was calculated for the 397 patients in the validation sample (overall mean score −0.23 ± 1.10), we noted similar results. For those who developed MAKE30 the mean was 0.27 ± 1.11 compared with −0.64 ± 0.94 in those who did not (mean difference 0.91 (95%CI 0.71–1.11); P < 0.001). The AROC was 0.74 (95%CI 0.69–0.79), and the Hosmer-Lemeshow test was not significant, indicating a similar level of performance observed in the training sample.

In addition, the addition of uNGAL to the multivariate model resulted in a significant increase (P < 0.001) in AROC in the validation dataset, compared to that predicted with uNGAL alone; AROC = 0.66 (95%CI 0.61–0.71) (Fig. 4B, Table 4).

### MAKE outcomes at 365 days

The overall MAKE365 incidence was 61.7%. The MAKE365 incidence after excluding the patients who already had MAKE30 was 32.4%; the additional endpoints were mortality (27.9%), RRT (2.7%), and persistent renal dysfunction (1.8%). Median urinary concentration between patients with MAKE365 and without MAKE365 were 625 (IQR 136, 2265) and 137 (93, 612) ng/mL, p < 0.001 (Fig. 2C).

**Figure 5:**
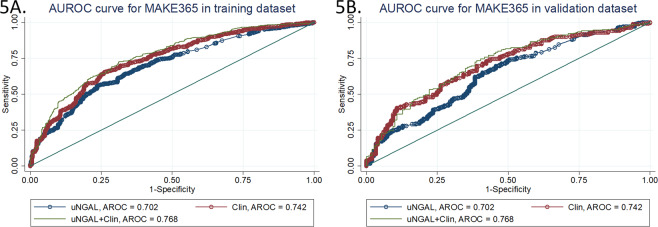
ROC curves from a multivariate clinical model, uNGAL, and the model with uNGAL for prediction of MAKE365 in the training dataset (**A**) and validation dataset (**B**).

Results from the multivariate equation for MAKE365 in the training dataset are shown in Table 3. An increased risk of MAKE365 was associated with increasing uNGAL concentration, increasing age, an ICU admission, AKI of stage 3 versus stages 1 and 2, sepsis, malignancy, ischemic heart disease, persistent AKI and chronic liver disease. Sex and ischemic cause of renal disease that were significant in the MAKE30 model were not significant in the MAKE365 model; however, age and ischemic heart disease that were not significant in the MAKE30 model were important in the MAKE365 model. The mean predicted MAKE365 score in the training sample was 0.60 ± 1.2 (1.0 ± 1.0 in those who developed MAKE365 and −0.07 ± 1.0 in those who did not; mean difference = 1.08 (95%CI 0.94–1.22; P < 0.001). The AROC was 0.77 (95%CI 0.74–0.80), which was significantly better than the AROC with uNGAL alone (0.70 (95%CI 0.67–0.74); P < 0.001) (Fig. 5A, Table 4). The Hosmer-Lemeshow test statistic was 4.73 (P = 0.79) (Table 5).

As found with the MAKE30 outcomes, the equation derived from the multivariate model in the training dataset also performed well in the validation dataset. The mean difference in MAKE365 scores between those with and without the outcome was 0.84 (95%CI 0.62–1.05); P < 0.001. The AROC was 0.72 (95%CI 0.67–0.77), suggesting reasonable discriminative ability. This was also a significant improvement (P < 0.001) on the AROC for uNGAL alone in predicting MAKE365 (AROC = 0.64 (95%CI 0.59–0.70) (Fig. 5B). The Hosmer-Lemeshow test statistic was 4.30 (P = 0.93), suggesting good calibration. The observed and expected numbers of MAKE365 events in the training and validation datasets is shown in Table 5.

### New-onset CKD and CKD progression

We further explored associations of uNGAL and new-onset CKD and CKD progression in patients who were alive at 1 year (n = 659). In patients without baseline CKD and were alive at 1 year (n = 370), there were a 68.9% incidence of new CKD (stage 3–5 and ESRD). Compared between transient AKI and persistent AKI, there were 69.1% vs 79.3% of patients who had CKD at 1 year (p = 0.006). Univariate and multivariate logistic regression revealed log_e_ uNGAL, age, and nephrotoxic cause of AKI to be associated with CKD (Supplementary Table 5). In patients with baseline CKD (n = 289), there was a 42.9% incidence of CKD progression. Univariate logistic regression revealed log_e_ uNGAL, persistent AKI, ischemic heart disease, AKI stage 3, and SCr at AKI diagnosis to be associated with CKD progression. Multivariate logistic regression, however, showed only uNGAL and ischemic heart disease as associated factors (Supplementary Table 6). In total, 18.2% of all patients progressed to ESRD.

## Discussion

To our knowledge, this is the first report using urine biomarker in a large cohort of heterogeneous patients for prediction of persistent AKI, MAKE30, and MAKE365. We found that uNGAL measured at the onset of AKI was associated with persistent AKI, MAKE30, MAKE365, new-onset CKD, and CKD progression. Urinary NGAL performed better than other markers for prediction of persistent AKI. For MAKE30 and MAKE365, predictive models combining uNGAL with clinical covariates significantly enhanced predictive value of MAKE30 and MAKE365 compared with uNGAL or clinical models alone.

Persistent AKI is associated with increased ICU length of stay, time on the ventilator, readmission, and mortality^8–10^. Persistent AKI also displayed significantly more adverse long-term outcomes with a cumulative incidence of>60% compared with 40% in transient AKI^26^. Coca *et al*. first described urinary injury markers as predictors for AKI duration in post-cardiac surgery patients^7^. Our data showed that patients with persistent AKI had higher uNGAL, uNGAL/Cr ratio, FENa, FEuric, and SCr than transient AKI. Among these biomarkers, uNGAL and uNGAL/Cr ratio were superior to other conventional biomarkers including SCr, by ROC curve analysis. These findings confirm the concept that an increase in creatinine lags for AKI diagnosis and adds little value in risk stratification. Thus, identification of persistent AKI by uNGAL is beneficial for timely intervention and more careful monitoring in-hospital and after discharge.

The 30-day and 1-year mortality rates in our study were 28.5% and 50%, similar to those reported in western cohorts^2,27–29^. Previous studies have shown uNGAL levels’ association with adverse in-hospital outcomes including mortality, which was likewise demonstrated in our study^18,30–35^. However, there is a paucity of data contributing to the evidence base regarding associations between uNGAL and long-term renal outcomes after AKI. Coca *et al*. reported the highest values of uNGAL and L-FABP were independently associated with long-term mortality in an AKI cohort^12^, but did not evaluate associations with renal function. Singer *et al*. showed that uNGAL measured at the time of AKI diagnosis was superior to creatinine levels and AKI staging in predicting long-term ESRD or death^23^. Our study included the largest number of patients to date, and showed an independent association of uNGAL with short-term and long-term death, ESRD, and persistent renal dysfunction. Furthermore, a few studies have investigated prognostic significance of uNGAL for progression of CKD in CKD cohorts^36–38^. In AKI patients, only one study explored uNGAL on ICU admission as a predictor CKD progression in MAKE-free ICU survivors^11^. Our study shows similar findings of uNGAL as an independent factor in AKI patients associated with new-onset CKD and CKD progression in 1-year survivors.

There have been few predictive models using urine biomarkers for prediction of AKI and AKI outcomes. Mcmahon *et al*. demonstrated the addition of urinary NGAL/albumin to the clinical model modestly improved the prediction of AKI, in particular severe stage 3 AKI and the prediction of 30-day RRT or death^24^. The TRIBE study showed that postoperative urine IL-18 and plasma NGAL improved AKI prediction over clinical model alone from AUC of 0.69 to 0.76 and 0.75, respectively^21^. In the SAPPHIRE and TOPAZ studies, a multivariate clinical model with TIMP2-IGFBP-7 helped predict moderate-to-severe AKI within 12 hours with AROC of 0.88^39^. Our study is the first to develop predictive model of MAKE30 and MAKE365 by using clinical models and uNGAL. The addition of uNGAL to clinical model significantly improved the risk prediction of MAKE30 and MAKE365 in both training and validation cohort. It must be emphasized that the clinical models alone performed better than uNGAL. The modest performance of uNGAL in our study maybe from the heterogeneous timing of uNGAL testing and presence of confounding factors including sepsis and CKD.

Our study has several limitations. First, because uNGAL is ordered at the discretion of any physician, the timing between AKI onset and uNGAL measurement may be heterogeneous. To mitigate this issue, we restricted inclusion in our study to those with NGAL results were collected within 72 hours of suspected AKI, and this reflects real world practice. Moreover, uNGAL request indications were predominantly for prediction of persistent AKI, implying that uNGAL tests were requested as early biomarkers for outcome prediction. Second, we did not have information of urine output, so outcomes might be underestimated by using creatinine criteria alone^40^. Third, we did not include patients without AKI, so the increased risk for adverse events with AKI staging was more prominent in AKI stage 3 compared with stage 1/2. Lastly, we used random split-sampling method for internal validation, so external validation of MAKE30 and MAKE365 predictive models is further needed. Despite these limitations, ours is a large observational study to assess the utility of NGAL for prognostic information in real-world practice, where physicians routinely use uNGAL to facilitate their clinical decisions. In addition, our study also evaluates NGAL measurements in the emergency room, general ward, and intensive care units, providing evidence on the usefulness of uNGAL in diverse clinical settings.

In conclusion, our current study demonstrates that significant association of uNGAL and risks for persistent AKI, MAKE30, and MAKE365. The use of clinical models incorporating AKI severity and other predictors with addition of uNGAL modestly improved prediction of MAKE30 and MAKE365. Determining uNGAL levels at the time of AKI diagnosis, in addition to improving diagnostic assessment, may help in early selection of patients who require close monitoring during hospital admission and after their stay, to prevent CKD progression, incident RRT, and death in AKI survivors.

## Materials and Methods

### Study design

The present study was conducted retrospectively on prospectively collected data of uNGAL registry at King Chulalongkorn Memorial Hospital. Inclusion criteria were patients aged ≥18 years with at least one uNGAL measurement within 72 hours of AKI onset between January 2016 and September 2017. Two nephrologists independently adjudicated acute kidney injury (AKI) status and onset using creatinine criteria from the KDIGO guideline without knowledge of NGAL value^41^. Patients were excluded if they had end-stage kidney disease, missing AKI status due to single measurement of serum creatinine, or unavailable outcomes at 365 days. The study protocol was approved by the Ethics Committee of Chulalongkorn University (IRB No. 037/61). Informed consent was waived by the Institutional Review Board of the Faculty of Medicine, Chulalongkorn University due to retrospective observational nature of the study and extraction of the patients’ data by an independent researcher with data deidentification. All research was performed in accordance with the international guidelines for human research protection as Declaration of Helsinki^42^, The Belmont Report^43^, and International Conference on Harmonization in Good Clinical Practice (ICH-GCP)^44^. The design, execution, and reporting of this study are in accordance with the Strengthening the Reporting of Observational Studies in Epidemiology (STROBE)^45^ and Transparent Reporting of a multivariable prediction model for Individual Prognosis or Diagnosis (TRIPOD) criteria^46^.

### Variables

Baseline characteristics of the patients were obtained from electronic medical records. AKI cause was adjudicated by two nephrologists by reviewing medical charts. Indication for uNGAL order was obtained from each physician at the time of request. Daily serum creatinine within the first week, at day 30, and at day 365 were obtained. For short-term outcomes, 30-day all-cause mortality, RRT, hospital length of stay, and renal recovery were recorded. For long-term outcomes, 365-day all-cause mortality, incident RRT, and readmission from all causes were collected. Mortality and renal replacement therapy were obtained from electronic in-house and national databases.

### Definition

AKI was defined and staged according to KDIGO criteria^41^. Baseline serum creatinine (SCr) was defined as the pre-admission SCr (obtained not more than 365 days prior) available from the hospital electronic records. When this value was not available, first admission SCr or the Modification of Diet in Renal Disease (MDRD) equation back-estimation was used, whichever one was lower^47,48^.

We defined transient AKI as an increase of SCr ≥1.5 times that of baseline within 7 days, that decreased to <1.5 times by 2 days after AKI onset. Persistent AKI was defined as an increase of SCr by 1.5 times of baseline that remained elevated after 3 days or more, or non-recovery before death^49^.

Major adverse kidney events at 30 days (MAKE30) comprised death, incident dialysis, and doubling of SCr within 30 days. Major adverse kidney events at 365 days (MAKE365) comprised death, incident dialysis, and persistent renal dysfunction (doubling of SCr or estimated glomerular filtration rate <50% from baseline) within 365 days. Renal recovery was defined as SCr <1.5 times of baseline and being free from RRT for 14 days^50^. Sepsis was diagnosed in accordance with the Third International Consensus Definitions for Sepsis and Septic Shock (SEPSIS-3)^51^. Chronic kidney disease (CKD) was defined as eGFR of less than 60 ml/min/1.73 m^2^ on at least 2 occasions lasting for more than 3 months. CKD stages were defined according to KDIGO guidelines and based on eGFR levels^52^. Progression of CKD stage in patients not on dialysis at study entry was determined by the last measured eGFR at 1 year in alive patients, resulting in reclassification to a more advanced stage or ESRD^37^.

### Urine chemistry and urinary NGAL

Urinary NGAL was measured at the time of AKI diagnosis by physicians’ discretion using a commercially available chemiluminescence method (Abbott, USA). Urinary Na, uric acid, and urea were also measured at the same time as uNGAL. The fractional excretion of sodium (FENa) was calculated by the formula (SCr × UNa)/(SNa × UCr). The fractional excretion of uric acid (FEuric) and fractional excretion of urea (FEurea) were calculated using the same formula.

### Outcomes

The primary outcome was predictive value of uNGAL for persistent AKI compared with conventional markers including serum creatinine, FENa, FEuric, and FEurea at the onset of AKI. The secondary outcomes were association and predictive performance of uNGAL alone and in combination with clinical models for MAKE30 and MAKE365. Association of uNGAL with new-onset CKD in patients without baseline CKD and CKD progression in patients with baseline CKD were also explored.

### Statistical analysis

Formal comparisons of continuous variables between groups were made by an unpaired t-test or by Mann–Whitney (Wilcoxon rank sum) test, and categorical data were compared using a Chi-Square or Fisher’s exact test, as appropriate. Univariate and multivariate logistic regression was to compare biomarkers associated with AKI persistence, new-onset CKD, and CKD progression, in the presence of clinical characteristics. We used logistic regression in preference to survival analysis techniques because there was no censoring in our dataset. Variables were adjusted for in the multivariate model if on univariate analysis they were related to persistent AKI at P < 0.1 using backwards stepwise procedure. AROC was compared between each biomarker for prediction of persistent AKI. Thereafter, we identified factors independently associated with MAKE30 and MAKE365 with multivariate regression models. For this purpose, the study cohort was randomized into a training dataset (n = 925) to generate a model, and validation dataset (n = 397) for assessing the adequacy of model fit. We used the training set to identify factors independently associated with MAKE30 and MAKE365, using a backwards stepwise procedure, and assessing Akaike’s information criteria (AIC) after each step. Model selection using AIC has better statistical properties compared to P-value based selection, and avoids arbitrary and inefficient selection rules based on P values^53^. Factors assessed in univariate models included uNGAL concentration, sex, age, AKI stage, persistent AKI, the ward in which the patient had the uNGAL sample collected, if the patient’s etiology of AKI was ischemic cause, and comorbidities including malignancy, diabetes, ischemic heart disease, chronic liver disease and sepsis. We assessed the linearity of continuous covariates against the logit function, and if this assumption was not met, the variable was transformed or modelled in quartiles. Adjacent categories were collapsed together if the odds ratios and 95%CI were similar. A logarithmic transformation was applied for uNGAL to linearize the covariate against the logit function.

We used the coefficients from the final multivariate models (the natural logarithms of the odds ratios) to derive scores for every individual that can be used to estimate the probability of developing MAKE30 and MAKE365. The score can be calculated by multiplying the coefficient by the corresponding covariate value for each individual. These MAKE30 and MAKE365 scores can be then used to estimate the probability of that individual developing a major adverse kidney event within 30 or 365 days, respectively, using the following equation:

$${\rm{Probability}}=\frac{{e}^{Score}}{1+\,{e}^{Score}}$$

Model adequacy was tested in several ways. First, we compared the mean scores in those who developed versus those who had not developed MAKE30 and MAK365 using an unpaired t-test. Second, we calculated the area under the Receiver Operating Characteristics Curve (AROC), as a measure of the ability of the model to discriminate between those with and without clinical outcomes^54^, comparing the AROC for uNGAL alone with that from the full multivariate model. Lastly, we assessed the calibration of the models in the training and validation datasets, by stratifying patients in deciles according to the MAKE30 and MAKE365 scores, and comparing the observed and expected number of events in each group using the Hosmer-Lemeshow Statistic^55^. Since the fit of any model is always better in the dataset used to derive the model than in the general population, we also calculated these statistics in the validation dataset to provide unbiased estimates of the model adequacy. Statistical analysis was conducted using Stata 16.0 (Statacorp, College Station, TX, USA).

## Supplementary information

Supplemental information.
