# Supplementary material for: Urine Neutrophil Gelatinase-associated Lipocalin (NGAL) for Prediction of Persistent AKI and Major Adverse Kidney Events
Source: Sci Rep. 2020 May 26;10:8718. doi: 10.1038/s41598-020-65764-w (PMC7250906; doi:10.1038/s41598-020-65764-w)
Supplement: Supplementary file 1 — Supplemental information. [file 41598_2020_65764_MOESM1_ESM.pdf]

Supplementary material

Supplementary Table 1 Indications for uNGAL request

Supplementary Table 2 Baseline characteristics in training and validation cohorts

Supplementary Table 3 Univariate and multivariate logistic regression analysis for MAKE30 in training dataset

Supplementary Table 4 Univariate and multivariate logistic regression analysis for MAKE365 in training dataset

Supplementary Table 5 Univariate and multivariate logistic regression analysis for new-onset CKD in patients without CKD at baseline and alive at 1 year

Supplementary Table 6 Univariate and multivariate logistic regression analysis for CKD progression in patients with baseline CKD and alive at 1 year

Supplementary Table 1 Indications for uNGAL request

**Indications[a]**	**N (%)**
Prediction of persistent AKI versus transient AKI	1,006 (76.1)
Prediction of RRT, death, or recovery	321 (24.3)
Differential diagnosis of hepatorenal syndrome and ATN	60 (4.5)
Antibiotic dose adjustment	7 (0.5)

[a]Seventy-two patients (5.4%) had more than one indication for uNGAL request.
Abbreviation AKI, acute kidney injury; RRT, renal replacement therapy; ATN, acute tubular necrosis

Supplementary Table 2 Baseline characteristics in training and validation cohorts

Variables	Training cohort (N=925)	Validation cohort (N=397)	Total (N=1,322)	*p* value
Age, y (mean ± SD)	65.4 ±17.5	67.4 ± 16.7	66 ± 17.3	0.06
Male (%)	509 (55.0)	217 (54.7)	726 (54.9)	0.9
Place of NGAL sent (%)				0.22
Ward	643 (69.7)	258 (65.3)	906 (68.5)	
ED	66 (7.2)	28 (7.1)	94 (7.1)	
ICU	213 (23.1)	109 (27.6)	322 (24.4)	
Admission diagnosis (%)				0.28
Cardiology	176 (19.3)	84 (21.4)	260 (19.9)	
Infection	202 (22.1)	101 (25.8)	303 (23.2)	
Malignancy	123 (13.5)	52 (13.3)	175 (13.4)	
Trauma/Surgery	44 (4.8)	18 (4.6)	62 (4.8)	
Rheumatology	21 (2.3)	7 (1.8)	28 (2.1)	
Hematology	43 (4.7)	9 (2.3)	52 (4.0)	
Endocrinology	19 (2.1)	7 (1.8)	26 (2.0)	
Ob-Gyn	3 (0.3)	2 (0.5)	5 (0.4)	
Gastroenterology	73 (8.0)	34 (8.7)	107 (8.2)	
Genitourinary system	55 (6.0)	21 (5.4)	76 (5.8)	
Respiratory system	136 (14.9)	43 (11.0)	179 (13.7)	
Others	18 (2.0)	14 (3.6)	32 (2.5)	
Admission type (%)				0.64
Medical	749 (82.0)	318 (81.1)	1,067 (81.8)	
Elective surgery	90 (9.9)	45 (11.5)	135 (10.3)	
Emergency surgery	74 (8.1)	29 (7.4)	103 (7.9)	
AKI staging (%)				0.26
1	318 (34.4)	148 (37.3)	466 (35.3)	
2	196 (21.2)	92 (23.2)	288 (21.8)	
3	411 (44.4)	157 (39.6)	568 (43.0)	
Sepsis (%)	400 (43.2)	172 (43.3)	572 (43.3)	0.98
Ischemic cause (%)	486 (52.7)	227 (57.2)	713 (54.0)	0.13
Nephrotoxic AKI (%)	89 (9.6)	29 (7.3)	118 (8.9)	0.17
CKD (%)	389 (42.1)	169 (42.7)	558 (42.2)	0.83
Comorbidities (%)				
Diabetes	329 (35.6)	152 (38.4)	481 (36.4)	0.33
Hypertension	476 (51.5)	215 (54.3)	691 (52.3)	0.35
Congestive heart failure			110 (8.3)	
Ischemic heart disease	68 (7.4)	42 (10.6)	58 (4.4)	0.05
Chronic liver disease	142 (15.4)	65 (16.4)	207 (15.7)	0.63
Baseline Cr, mg/dL	1.5 (1.1,2.5)	1.5 (1.0,2.5)	1.5 (1.0, 2.5)	0.59
Baseline GFR, mL/min/1.73m^2	46.3 (27.4, 71.6)	45.0 (28.7, 67.4)	45.7 (28.0, 70.6)	0.43

Cr at AKI diagnosis, mg/dL	2.3 (1.6, 3.5)	2.2 (1.6, 3.2)	2.4 (1.7, 3.5)	0.03
Peak creatinine at day 30, mg/dL	2.9 (1.9, 4.4)	2.7 (1.9, 4.1)	2.9 (1.9, 4.3)	0.12
Last GFR at day 30, mL/min/1.73m^2	43.1 (27.7, 67.2)	43.6 (27.3, 65.3)	43.4 (27.5, 65.7)	0.96
Urine NGAL, ng/mL	356 (102,1500)	418 (102,1460)	374 (102, 1500)	0.72
Urine NGAL-Cr ratio, ng/mg	6.1 (1.7, 35.6)	5.3 (1.8, 28.6)	5.5 (1.8, 31.8)	0.58
FENa, percent	1.7 (0.6, 3.9)	1.7 (0.65, 4.36)	1.85 (0.6, 5.2)	0.62
FEUric, percent	11.8 (5.9, 20.7)	12.55 (6.7, 22.7)	12 (6.3, 21.6)	0.37
FEUrea, percent	39.98 (26.6, 57.2)	37.7 (23.6, 56.5)	38.4 (24.0, 50.8)	0.56

* Values are presented as median (IQR)

Abbreviations NGAL, neutrophil gelatinase-associated lipocalin; FENa, fractional excretion of sodium; FEuric, fractional excretion of uric; FEurea, fractional excretion of urea; Cr, creatinine; AKI, acute kidney injury; SD, standard deviation; ED, emergency department; ICU, intensive care unit; CKD, chronic kidney disease; GFR, glomerular filtration rate

Supplementary Table 3 Univariate and multivariate logistic regression analysis for MAKE30 in the training dataset

	Univariate analysis		Multivariate analysis	
	OR (95% CI)	P value	Adjusted OR (95% CI)	P value
Log uNGAL	1.65 (1.51-1.81)	**<0.001**	1.47 (1.32 - 1.63)	**<0.001**
Age (per 10 years)	1.01 (0.94-1.09)	0.74	-	-
Male (versus female)	1.39 (1.07-1.80)	**0.01**	1.31 (0.97 - 1.77)	0.08
ICU (versus other wards)	2.16 (1.58-2.95)	**<0.001**	1.88 (1.33 - 2.67)	**<0.001**
AKI stage				
1	Reference			
2	1.32 (0.91-1.93)	0.15	Reference (stage 1 & 2)	
3	3.47 (2.54-4.73)	**<0.001**	2.35 (1.73 - 3.21)	**<0.001**
Sepsis	1.89 (1.45-2.46)	**<0.001**	1.47 (1.04 - 2.08)	**0.03**
Ischemic AKI	0.77 (0.59-1.00)	**0.05**	1.37 (0.98 - 1.92)	**0.06**
Malignancy	2.02 (1.37-2.98)	**<0.001**	1.85 (1.18 - 2.9)	**0.007**
Ischemic heart disease	1.63 (0.84-3.17)	0.15	-	-
Persistent AKI	1.99 (1.50-2.63)	**<0.001**	0.95 (0.43 - 2.09)	0.89
Chronic liver disease	1.40 (0.98-2.00)	0.07	1.48 (0.98 - 2.23)	0.06

Abbreviations OR, Odds ratio; CI, confidence interval; uNGAL, urine neutrophil gelatinase-associated lipocalin; ICU, intensive care unit; AKI, acute kidney injury; Cr, creatinine

Supplementary Table 4 Univariate and multivariate logistic regression analysis for MAKE365 in the training dataset

	Univariate analysis		Multivariate analysis	
	OR (95% CI)	P value	Adjusted OR (95% CI)	P value
Log uNGAL	1.60 (1.45-1.75)	**<0.001**	1.41 (1.26 - 1.57)	**<0.001**
Age (per 10 years)	1.14 (1.05-1.23)	**0.001**	1.15 (1.06 - 1.26)	**0.001**
Male (versus female)	1.22 (0.93-1.59)	0.15	-	-
ICU (versus other wards)	1.60 (1.15-2.23)	**0.005**	1.35 (0.93 - 1.97)	0.113
AKI stage				
1	Reference			
2	0.86 (0.60-1.23)	0.41	Reference (stage 1&2)	
3	2.27 (1.66-3.09)	**<0.001**	1.69 (1.21 - 2.34)	**0.002**
Sepsis	1.97 (1.49-2.60)	**<0.001**	1.43 (1.03 - 1.98)	**0.03**
Ischemic AKI	0.80 (0.61-1.05)	0.10	-	
Malignancy	2.10 (1.36-3.25)	**0.001**	2.13 (1.3 - 3.48)	**0.003**
Ischemic heart disease	4.01 (1.54-10.40)	**0.004**	6.69 (2.41 - 18.56)	**<0.001**
Persistent AKI	2.48 (1.87-3.31)	**<0.001**	1.26 (0.6 - 2.64)	0.54
Chronic liver disease	1.69 (1.14-2.50)	**0.009**	2.14 (1.38 - 3.34)	**0.001**

Abbreviations OR, odds ratio; CI, confidence interval; uNGAL, urine neutrophil gelatinase-associated lipocalin; ICU, intensive care unit; AKI, acute kidney injury; Cr, creatinine

Supplementary Table 5 Univariate and multivariate logistic regression analysis for new-onset CKD in patients without CKD at baseline and alive at 1 year

	Univariate analysis		Multivariate analysis	
	OR (95% CI)	P value	OR (95% CI)	P value
Log uNGAL	1.18 (1.02,1.36)	**0.02**	1.17 (1.01,1.36)	**0.04**
Age (per 10 years)	1.03 (1.02,1.05)	**<0.001**	1.03 (1.02,1.05)	**<0.001**
Male (versus female)	1.30 (0.83,2.05)	0.25	-	-
ICU (versus other wards)	1.26 (0.73,2.16)	0.41	-	-
Ischemic AKI	0.96 (0.62,1.50)	0.86	-	-
Nephrotoxic AKI	2.62 (1.13,6.07)	**0.03**	2.74 (1.15,6.53)	**0.02**
Sepsis	1.05 (0.66,1.65)	0.85	-	-
Cr at AKI diagnosis	0.99 (0.90,1.09)	0.86	-	-
AKI stage			-	-
1	Reference			
2	0.83 (0.46,1.49)	0.52		
3	0.79 (0.47,1.34)	0.38		
Persistent AKI	1.19 (0.75,1.90)	0.46	-	**-**

Abbreviations OR, odds ratio; CI, confidence interval; uNGAL, urine neutrophil gelatinase-associated lipocalin; ICU, intensive care unit; Cr, creatinine; CKD, chronic kidney disease; AKI, acute kidney injury

Supplementary Table 6 Univariate and multivariate logistic regression analysis for CKD progression in patients with baseline CKD and alive at 1 year

	Univariate analysis		Multivariate analysis	
	OR (95% CI)	P value	OR (95% CI)	P value
Log uNGAL	1.32 (1.12,1.56)	**0.001**	1.23 (1.02,1.47)	**0.02**
Age (per 10 years)	0.99 (0.98,1.01)	0.46	-	-
Male (versus female)	1.24 (0.78,1.98)	0.37	-	-
ICU (versus other wards)	1.17 (0.64,2.13)	0.62	-	-
Ischemic heart disease	4.07 (1.43,11.63)	**0.009**	4.56 (1.50,13.84)	**0.007**
Sepsis	0.87 (0.53,1.45)	0.60	-	-
CKD stage			-	-
3	Reference	Ref		
4	0.93 (0.55,1.58)	0.78		
5	1.26 (0.60,2.66)	0.55		
Ischemic AKI	1.34 (0.83,2.17)	0.23	-	-
Nephrotoxic AKI	1.03 (0.43,2.42)	0.95	-	-
Cr at AKI diagnosis	1.16 (1.03,1.30)	**0.01**	1.04 (0.91,1.20)	0.56
AKI stage				
1	Reference	Ref	Reference	
2	0.73 (0.37,1.45)	0.37	(stage 1&2)	
3	1.90 (1.13,3.20)	**0.02**	1.30 (0.69,2.44)	0.42
Persistent AKI	2.74 (1.42,5.30)	**0.003**	1.84 (0.86,3.93)	0.07

Abbreviations OR, odds ratio; CI, confidence interval; uNGAL, urine neutrophil gelatinase-associated lipocalin; ICU, intensive care unit; Cr, creatinine; CKD, chronic kidney disease; AKI, acute kidney injury
